# Comparative analysis of apoptotic changes in peripheral immune organs and lungs following experimental infection of piglets with highly pathogenic and classical porcine reproductive and respiratory syndrome virus

**DOI:** 10.1186/1743-422X-11-2

**Published:** 2014-01-06

**Authors:** Gang Wang, Yuli He, Yabin Tu, Yonggang Liu, En-Min Zhou, Zifeng Han, Chenggang Jiang, Shujie Wang, Wenda Shi, Xuehui Cai

**Affiliations:** 1 State Key Laboratory of Veterinary Biotechnology, Harbin Veterinary Research Institute, Chinese Academy of Agricultural Sciences, Harbin, Heilongjiang Province, 150001, PR China; 2 Department of Preventive Veterinary Medicine, College of Veterinary Medicine, Northwest A&F University, Yangling, Shaanxi, 712100, PR China; 3 Northeast Agricultural University, Harbin, 150030, PR China

**Keywords:** Highly pathogenic PRRSV, Classical PRRSV, Peripheral immune organs, Lung, Cell apoptosis

## Abstract

**Background:**

Our previous studies have demonstrated that piglets infected with highly pathogenic porcine reproductive and respiratory syndrome virus (HP-PRRSV) may develop significant thymus atrophy, which related to thymocytes apoptosis. However, apart from that detected in the thymus, there are no reports describing cell apoptosis induced by HP-PRRSV infection. In this study, we analyzed comparatively the pathological changes, cell apoptosis and viral load in peripheral immune organs including tonsil, inguinal lymph nodes (ILNs) and spleen and lungs following experimental infection of piglets with HP-PRRSV HuN4 and classical PRRSV CH-1a.

**Findings:**

HP-PRRSV HuN4 exhibited much stronger cell tropism than CH-1a in immune organs and lungs of piglets. HuN4 infection led to the serious injuries in tonsils, ILNs, spleens and lungs, especially apoptosis in these organs was significant.

**Conclusions:**

HuN4 infection induced severe lesions (gross pathology, histopathology and cell apoptosis) in the peripheral immune organs and lungs of infected piglets. Large numbers of apoptotic cells in immune organs and lung induced by HuN4 may play a role in the pathogenesis of the HP-PRRS and the distinct injuries caused by HuN4 infection may be associated with the high mortality rate of HP-PRRS in pigs.

## Findings

### Summary and experimental design

Porcine reproductive and respiratory syndrome (PRRS) was first recognized in the United States and Europe in 1987 and 1990, respectively [1-4]. Highly pathogenic PRRS (HP-PRRS) first emerged in China and Vietnam in 2006, and then spread rapidly in pigs throughout Asia [5,6]. Classical PRRSV infection induces apoptosis in different organs (lungs, testes, lymph nodes and thymus) in vivo and in cell lines in vitro [7-10], with apoptosis observed in both PRRSV-positive and PRRSV-negative cells [9,11]. However, apart from that detected in the thymus, there are no reports describing cell apoptosis induced by HP-PRRSV infection.

PRRSV CH-1a (GenBank accession no. EU807840) and HuN4 (GenBank accession no. EF635006) represent the classical and highly pathogenic PRRSV strains, respectively. Our previous studies have demonstrated that piglets infected with the HuN4 strain developed significant thymus atrophy, which was related to cell apoptosis; however, piglets infected with the CH-1a strain showed mild pathological lesions and a few apoptotic cells in the thymus [12,13]. In this study, we sought to clarify the role of apoptosis in lesions of the peripheral immune organs and lungs of piglets experimentally infected with either the highly pathogenic (HuN4) or the classical (CH-1a) virulence strains of PRRSV. The experimental design has been described in previously published study [13]. Briefly, three groups (15 piglets/group) were used in this study, Group 1 and Group 2 piglets were inoculated with 2 ml of HuN4 and CH-1a (7.9 × 10^5^ TCID_50_ in 2 ml DMEM medium), respectively. Group 3 were sham-inoculated with 2 ml of DMEM medium. Three piglets from each group were humanely euthanized at 0 day post-inoculation (DPI), 3 DPI, 7 DPI, 10 DPI and14 DPI, respectively. In present study, we utilized the tonsils, inguinal lymph nodes (ILNs), spleens and lungs of piglets from the published study and this study is an extension of that study.

### Gross pathology

The gross pathological changes of peripheral immune organs and lungs were recorded during necropsy. In Group 1, the lesions in tonsil, ILNs and spleen were characterized by swelling or diffuse hemorrhage, which were the same to our previous reported [12], and the lungs showed red and beige variegated appearance from 7 DPI [14]. In Group 2, the main lesions were found in the ILNs and lungs and were characterized by swelling in the ILNs and mild interstitial pneumonia in the lungs. In Group 3 piglets, the organs investigated remained normal throughout the experiment and no lesions were detected.

### Detection of virus in tissues

Real-time RT-PCR quantitation (qRT-PCR) of viral loads in peripheral immune organs and lungs was performed as previously described [14]. In Group 1, high titers of PRRSV were detected in tonsils, ILNs, spleen and lungs of three piglets from 3 DPI to the end of the experiment (approximately 10^8^ copies/g), which were similar to those observed in previous study [12]. In Group 2, PRRSV was also detected in tonsils, ILNs, spleen and lungs of piglets euthanized at 3 DPI. However, the viral loads were low (approximately 10^5^ copies/g) and were maintained at low viral loads to 14 DPI. No viruses were detected in any of the tissues investigated from Groups 1 and 2 euthanized at 0 DPI or from Group 3 piglets euthanized at any of the time-points during the experiment.

### Histopathologic examination

Tissue samples of the tonsil, ILNs, spleen and lungs collected on different DPI were used for histological examination using hematoxylin and eosin (H&E) staining as described previously [15]. Tonsils, ILNs and spleens were evaluated for the presence of lymphoid depletion ranging from 0 (normal) to 3 (severe) and inflammation and replacement of follicles ranging from 0 (normal) to 3 (severe) according to the established scoring system [16-18]. Lung sections were scored for the presence and severity of interstitial pneumonia ranging from 0 (no microscopic lesions) to 4 (severe interstitial pneumonia) as described previously [19]. In Group 1, lymphoid depletion, inflammation and replacement of follicles of tonsils, ILNs and spleens appeared at 3 DPI, and became more severe at 7 DPI; severe interstitial pneumonia of the lungs were observed at 10 DPI (Table 1). In Group 2, lymphoid depletion, inflammation and replacement of follicles and interstitial pneumonia were mild throughout the experiment.

**Table 1:** Histopathological changes of immune organs and lungs at different days post-inoculation (DPI)

**Group**	DPI^*^	**Histological lesion score**^**# **^**(No. of piglets with lesions/no. of piglets examined)**						
		**Tonsil**		**ILN**		**Spleen**		**Lung**
		**a**	**b**	**a**	**b**	**a**	**b**	**c**
1	0	0	0	0	0	0	0	0
	3	1 (1/3)	0	1 (2/3)	1 (1/3)	1 (2/3)	0	1 (1/3)
	7	2 (3/3)	1 (1/3)	1.5 (2/3)	1 (2/3)	1 (2/3)	1.5 (2/3)	1 (2/3)
	10	3 (3/3)	2.7 (3/3)	1.7 (3/3)	1.7 (3/3)	2 (3/3)	2 (2/3)	3 (3/3)
	14	3 (3/3)	3 (3/3)	3 (3/3)	2 (3/3)	3 (3/3)	2 (3/3)	4 (3/3)
2	0	0	0	0	0	0	0	0
	3	0	0	0	0	0	0	0
	7	2 (1/3)	0	1 (2/3)	0	0	1 (1/3)	1 (1/3)
	10	2 (2/3)	1.3 (3/3)	1 (2/3)	0	1 (2/3)	0	1.3 (3/3)
	14	1 (1/3)	1 (2/3)	1 (2/3)	1 (2/3)	1 (2/3)	1.5 (2/3)	1.3 (2/3)

^*^ Days post-inoculation when pigs were necropsied.

^#^ Mean histological lesion score of piglets with lesions.

a Histological lymphoid depletion score: 0, normal; 1, mild; 2, moderate; 3, severe.

b Inflammation and replacement of follicles score: 0, normal; 1, mild; 2, moderate; 3, severe.

c Interstitial pneumonia score: 0, no microscopic lesions; 1, mild interstitial pneumonia; 2, moderate multifocal interstitial pneumonia; 3, moderate diffuse interstitial pneumonia; 4, severe interstitial pneumonia.

### Cell apoptosis detection

Apoptotic cells were detected in tonsils, ILNs, spleen and lungs using the TUNEL technique as previously reported [20] with the In Situ Cell Death Detection Kit (Roche, Mannheim, Germany) according to the manufacturer’s instructions. The number of labeled cells per unit area (mm^2^) was calculated and the results were expressed as mean ± standard deviation. Evaluation of apoptotic thymocytes was conducted as our previously described [13]*.* Statistical analyses were performed using GraphPad PRISM software for analysis of variance (ANOVA), and the P-value <0.05 was considered statistical significance.

**Figure 1:**
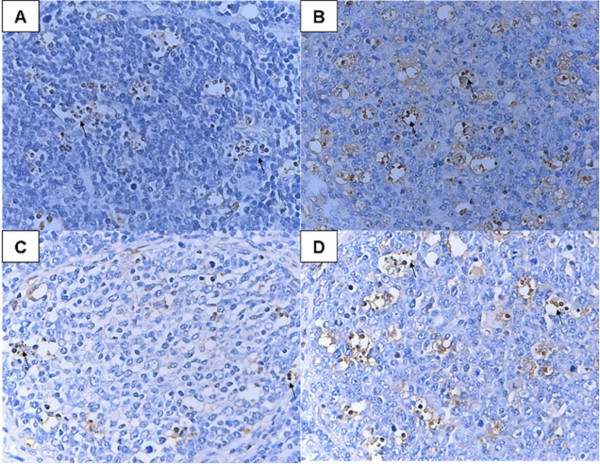
**TUNEL assay with tonsil tissues of piglets during infection.** Shown are tonsil tissue sections from HuN4 strain-infected piglets at 3 DPI **(A)** and 10 DPI **(B)** and from CH-1a strain-infected piglets at 3 DPI **(C)** and 10 DPI **(D)**, respectively. The arrows denote areas of TUNEL-positive cells. Cells undergoing apoptosis were mainly lymphocytes.

In Group 1, TUNEL-positive cells were observed in tonsils at 3 DPI and peaked at 10 DPI, and most of the apoptotic cells were observed in lymphatic nodules (Figure 1A and B), the similar results were observed in ILNs and spleen. In Group 2, TUNEL-positive cells were also observed during the experiment although, at lower frequencies than that of Group 1 at 3, 7 and 10 DPI ( *P* < 0.05) (Table 2).

**Table 2:** The number of apoptotic cells in immune organs and lungs at different days post-infection (DPI)

DPI^*^	**The number of apoptotic cells (per mm**^**2**^**)**											
	**Tonsil**			**ILN**			**Spleen**			**Lung**		
	**1**	**2**	**3**	**1**	**2**	**3**	**1**	**2**	**3**	**1**	**2**	**3**
0	0.4 ± 0.2	0.5 ± 0.2	0.4 ± 0.2	0.4 ± 0.2	0.3 ± 0.2	0.4 ± 0.2	0.4 ± 0.2	0.2 ± 0.1	0.3 ± 0.2	0.4 ± 0.2	0.4 ± 0.2	0.3 ± 0.2
3	17.7 ± 0.9^a^	5.0 ± 0.7 ^b^	0.3 ± 0.2^c^	18.3 ± 0.8^a^	6.3 ± 0.5^b^	0.4 ± 0.2^c^	8.1 ± 0.5^a^	4.3 ± 0.5^b^	0.4 ± 0.2^c^	8.2 ± 0.8^a^	2.8 ± 0.5^b^	0.4 ± 0.2^c^
7	20.0 ± 0.8^a^	7.7 ± 0.4^b^	0.4 ± 0.2^c^	21.5 ± 0.6^a^	9.0 ± 0.5^b^	0.4 ± 0.2^c^	13.0 ± 0.8^a^	7.3 ± 0.4^b^	0.4 ± 0.2^c^	8.7 ± 0.7^a^	4.4 ± 0.6^b^	0.4 ± 0.2^c^
10	21.9 ± 1.4^a^	11.0 ± 0.8^b^	0.5 ± 0.2^c^	23.8 ± 0.8^a^	13.6 ± 0.5^b^	0.4 ± 0.2^c^	19.4 ± 0.9^a^	9.5 ±0.4^b^	0.5 ± 0.2^c^	9.0 ± 0.4^a^	5.6 ± 0.6^b^	0.4 ±0.2^c^
14	14.8 ± 0.8^a^	12.6 ± 0.8^a^	0.2 ± 0.1^b^	14.2 ± 0.8^a^	10.4 ± 0.7^b^	0.4 ± 0.2^c^	14.2 ± 0.6^a^	13.9 ± 0.8^a^	0.4 ± 0.2^b^	6.8 ± 0.5^a^	6.1 ± 0.4^a^	0.3 ± 0.2^b^

*Days post-inoculation when pigs were necropsied.

^a,b,c^Values with different superscripts indicate mean value score differences among groups (*P* < 0.05).

**Figure 2:**
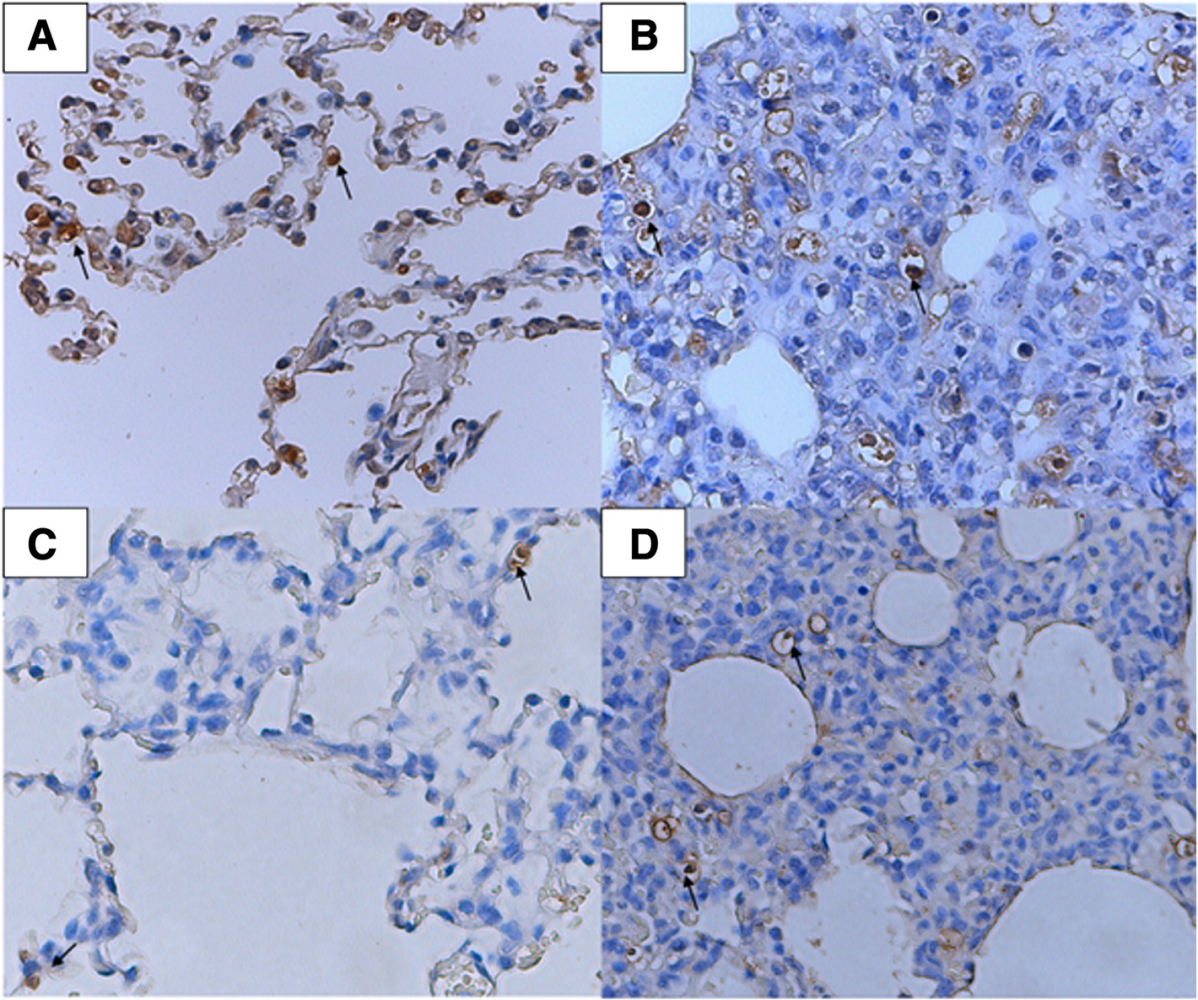
**TUNEL assay with lung tissues of piglet during infection.** Shown are lung tissue sections from HuN4 strain-infected piglets at 3 DPI **(A)** and 10 DPI **(B)** and from CH-1a strain-infected piglets at 3 DPI **(C)** and 10 DPI **(D)**, respectively. The arrows in **A** and **C** show cells undergoing apoptosis were type II pneumocytes, and in **B** and **D** were macrophages.

The number of TUNEL-positive cells in the lungs of Group 1 was approximately 9 cells per mm^2^ from 3 to 10 DPI (Figure 2), and the apoptotic cells included the porcine alveolar macrophages (PAMs) and type II pneumocytes (Figure 2A and B). In Group 2, this number increased from 3 to 6 cells per mm^2^ during the period from 3 to 14 DPI, type II pneumocytes and macrophages (Figure 2C and D) were also observed undergoing apoptosis. A significant difference in the number of apoptotic cells among groups was detected at 3, 7 and 10 DPI (*P* < 0.05) (Table 2).

## Discussion

HP-PRRSV infection induces higher rates of morbidity and mortality in animals of different ages than PRRSV infection [5,6,21,22]. However, differences in the lesions on peripheral immune organs and lungs following experimental infection of pigs with highly pathogenic and classical PRRSV have not yet to be reported. In this study, higher viral loads were detected in peripheral immune organs (tonsils, ILNs, spleens) and lungs of piglets infected with the HP-PRRSV HuN4 strain than that of pigs infected with the classical PRRSV CH-1a strain. Viral replication in these organs leads to the serious lesions (Table 1). Depletion of peripheral immune organs adversely affects the capacity for eradication of viruses and bacteria, and immunity to endogenous and exogenous substances with a variety of specific morphological and functional consequences. HuN4 infection causes severe immunosuppression and enhances susceptibility to secondary infections and also induces severe thymic atrophy [13]. The severe damage to immune organs and lungs mediated by HuN4 may cause the high mortality rate associated with HP-PRRS in pigs.

Besides the apoptosis observed in thymocytes [13], significant numbers of apoptotic cells were also observed in tonsils, ILNs, spleen and lungs of piglets infected with HuN4 (Table 2). However, few apoptotic cells were detected in these tissues of piglets infected with CH-1a, which was consistent with the results of previous studies of cell apoptosis induced by classical PRRSV infection [10,23,24]. Large numbers of apoptotic cells in these organs induced by HuN4 infection indicated that apoptosis may play a role in the pathogenesis of the HP-PRRS, with increased apoptotic cells associated both with more severe tissue injury and with increased viral loads. Moreover, more apoptotic cells were observed in immune organs of HP-PRRSV infected piglets pointing out a mechanism which might play a role in the induction of the erratic host immune response observed in HP-PRRSV infected pigs [14]. The apoptotic cells, along with the subsequent microscopical and gross lesions may trigger events that immune balance and induce immunosuppression. However, a specific association between cell apoptosis and PRRSV infection requires further investigation.

In conclusion, this is the first report describing a comparative investigation of apoptotic cells in peripheral immune organs and lungs of piglets following experimental infection with the highly pathogenic and classical PRRSV strains. Large numbers of apoptotic cells in immune organs and lung induced by HuN4 infection may play a role in the pathogenesis of the HP-PRRS.

## Competing interests

The authors declare that they have no competing interests.

## Authors’ contributions

Conceived and designed the experiments: CXH and WG; Performed the experiments: WG, HYL, LYG, HZF, JCG, WSJ and SWD. Critical discussion and final approval of manuscript: WG, HYL, TYB, LYG, ZEM, HZF, JCG, WSJ, SWD, CXH. All authors approved the final manuscript.
